# The Role of TSLP and IL-1 β and Their Genetic Variants in the Pathogenesis of Single and Multiple Atopic Diseases in Children

**DOI:** 10.3390/jcm14020598

**Published:** 2025-01-17

**Authors:** Hanna Sikorska-Szaflik, Anna Dębińska, Joanna Połomska, Anna Drabik-Chamerska, Barbara Sozańska

**Affiliations:** Department and Clinic of Paediatrics, Allergology and Cardiology, Wroclaw Medical University, ul. Chałubińskiego 2a, 50-368 Wrocław, Poland; anna.debinska@umw.edu.pl (A.D.); joanna.polomska@umw.edu.pl (J.P.); anna.drabik-chamerska@umw.edu.pl (A.D.-C.); barbara.sozanska@umw.edu.pl (B.S.)

**Keywords:** TSLP, IL-1β, polymorphism, atopy

## Abstract

Allergic diseases commonly coexist, manifesting in a sequence described as the “allergic march”. **Background/Objectives:** This study aimed to evaluate TSLP’s and IL-1β’s potential as biomarkers in both single and multi-pediatric atopic diseases like atopic eczema, food allergy, and anaphylaxis and analyze specific SNPs in the TSLP and IL-1β genes to determine their associations with their occurrence and severity. **Methods:** This analysis included 109 atopic children diagnosed with atopic dermatitis, food allergy, or anaphylaxis alongside a control group of 57 non-atopic children. Recruitment was facilitated through the use of a comprehensive questionnaire. For the study population, the allergen profile was characterized at the molecular level by measuring specific IgE to purified natural or recombinant allergens, assessing serum levels of circulating TSLP and IL-1β, and identifying single-nucleotide polymorphisms in TSLP (rs2289277) and IL-1β (rs16944 C-511T). **Results:** The serum levels of TSLP and IL-1β were elevated in the study groups compared to the control group, highlighting their significance in the pathogenesis of the studied diseases. Carrying a higher number of the risk allele [C] in the TSLP SNP rs2289277 is associated with the greatest likelihood of having multiple concurrent allergic conditions, with the highest risk observed in individuals with all three conditions—atopic dermatitis, food allergy, and anaphylaxis, simultaneously. Moreover, children carrying the risk allele had a twofold increased risk of polysensitization, which rose to sixfold in those with two copies of the risk allele. Although no significant variations in genotype frequencies were detected for IL-1β rs16944, significant associations were observed for TSLP rs2289277, particularly with conditions such as atopic dermatitis, food allergy, anaphylaxis, and combinations of these diseases. **Conclusions:** Further research is required to elucidate these pathways and their role in the development of allergic diseases.

## 1. Introduction

The prevalence of allergic diseases has been increasing globally over the past few decades, posing a significant public health challenge. This rise in conditions such as atopic dermatitis (AD), food allergy (FA), asthma, and allergic rhinitis is particularly pronounced in industrialized countries, likely influenced by factors such as environmental changes, urbanization, dietary shifts, and reduced microbial exposure due to improved hygiene practices [1]. Allergic diseases frequently coexist, often manifesting in a sequence described as the “allergic march”. This progression—from eczema to food allergies, asthma, and allergic rhinitis—highlights shared pathophysiological pathways influenced by genetic and environmental factors. Atopic dermatitis, a chronic inflammatory skin disease characterized by itchy, red, and dry skin lesions, is one of the most common allergic conditions. It impacts approximately 10% of adults and 20% of children globally, with 60% of cases manifesting within the first year of life [2,3].

The pathogenesis of AD is multifactorial, involving genetic predisposition, environmental influences, and immune dysregulation. Notably, AD is a key risk factor for FA development. It has been suggested that allergen sensitization through impaired skin barriers in AD patients plays a critical role in initiating food sensitization and food allergies [4,5]. This connection exemplifies how allergic diseases amplify the other’s progression. Food allergy, in turn, can lead to life-threatening anaphylactic reactions, the most severe manifestation of an allergic response. Anaphylaxis is a systemic hypersensitivity reaction triggered by food allergens, which can cause respiratory distress, cardiovascular compromise, and even death. Its rising prevalence further highlights the urgency of understanding and addressing allergic diseases as interconnected phenomena [6].

Among the molecular drivers of these allergic conditions are cytokines such as thymic stromal lymphopoietin (TSLP) and interleukin-1 beta (IL-1β). TSLP, primarily produced by epithelial cells in the skin, lungs, and gastrointestinal tract, plays a pivotal role in initiating and sustaining allergic inflammation. It activates dendritic cells, which in turn drive naive T cells towards a Th2 phenotype, promoting the production of cytokines like IL-4, IL-5, and IL-13 [7]. These cytokines contribute to hallmark allergic responses, including eosinophil activation, IgE production, and mucus secretion [8]. Environmental factors such as epithelial damage, microbial exposure, and viral infections can elevate TSLP levels, intensifying allergic inflammation. Elevated TSLP has been implicated in multiple allergic conditions, including AD, asthma, allergic rhinitis, and FA, making it a promising therapeutic target [8].

Similarly, IL-1β, a potent pro-inflammatory cytokine, amplifies inflammation by promoting the recruitment and activation of immune cells and also plays a role in the regulation of the skin barrier by modulating the expression of genes involved in keratinocytecyte differentiation and tight junctions. IL-1β can promote the recruitment and activation of various immune cells, such as neutrophils and macrophages, and enhance the production of other cytokines, contributing to the inflammatory condition. Elevated levels of IL-1β have been observed in various allergic diseases, where it contributes to tissue damage, chronicity, and exacerbations. For instance, IL-1β plays a role in the severity of conditions like AD, asthma, and allergic rhinitis, as well as in the immune dysregulation leading to food allergies [9,10,11,12,13]. We hypothesized that, although IL-1β is not traditionally associated with Th2-driven inflammation in AD, it could still contribute to disease progression through its effects on skin barrier dysfunction and early immune responses, especially in the context of co-morbid allergic conditions.

Polymorphic variations in interleukin genes may modulate their expression, potentially contributing to an increased susceptibility to allergic diseases. Single nucleotide polymorphisms (SNPs) in genes encoding TSLP and IL-1β have been associated with variations in cytokine expression and function [14,15,16,17]. These genetic variations may predispose individuals to allergic conditions and influence the severity and co-occurrence of diseases such as AD and FA. However, the relationship between these genetic factors, cytokine levels, and the presence of multiple allergic diseases remains poorly understood.

The objective of this research was to investigate the concentrations of TSLP and IL-1β in children with atopic eczema, FA, and anaphylaxis to healthy controls, assessing their potential as biomarkers for allergic conditions. We also explored whether elevated levels of these cytokines correlate with the presence of multiple allergic diseases in a single patient and/or the sensitization profile. Furthermore, we examined specific SNPs in the TSLP and IL-1β genes to determine their association with the occurrence and number of coexisting allergic diseases, addressing the gaps in understanding the genetic and immunological mechanisms underlying allergic multimorbidity.

## 2. Materials and Methods

### 2.1. Study Design and Ethical Approval

The presented observational, case–control study was conducted at the Wroclaw Medical University under project number SUBZ.A220.23.019. The protocol of this study, a written description of the study prepared for the study participants, and consent forms for participation in the study were approved by the Ethics Committee of the Wroclaw Medical University, Poland (protocol code 47/2023, 18 January 2023, Wroclaw). This research was performed in accordance with the Declaration of Helsinki for research involving human participants. The participants and their guardians were informed that they retained the right to discontinue their participation at every stage of the research. Written informed consent forms for participation in the study, containing consent for genetic analyses, were obtained from study participants and their legally authorized representatives before the laboratory procedures began.

### 2.2. Enrollment and Study Procedures

**Figure 1:**
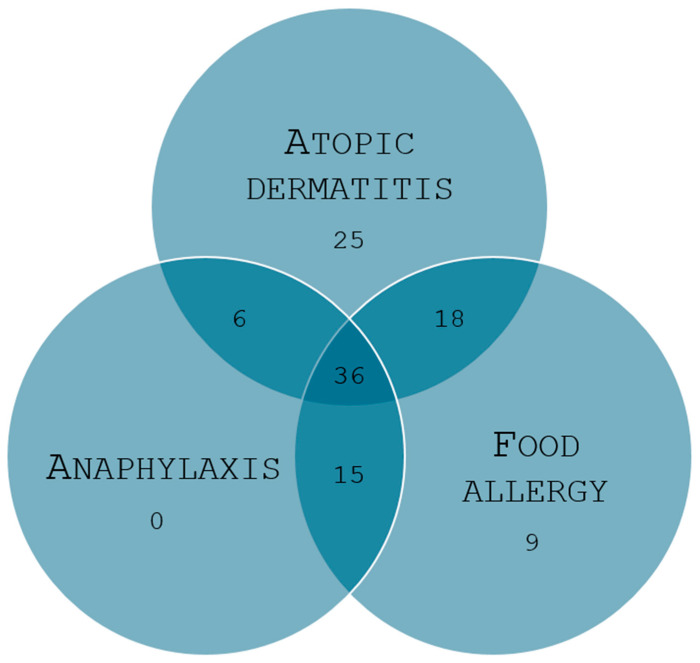
Distribution of subgroup sizes in the study population.

Participants included in the study were European Caucasians (n = 166) aged six months to eighteen years at the time of recruitment. The study cohort was randomly selected from patients at the Pediatric and the Allergology Departments and the Pediatric Allergology Outpatient Clinic at Wroclaw Medical University Hospital, Poland. All participants underwent specific IgE (sIgE) testing using the Allergy Explorer Kit ALEX 2 (Cat. No. Macro Array Diagnostics, Vienna, Austria), and each participant was provided with an original sociodemographic and clinical questionnaire. Based on the sIgE results and questionnaire data, individuals were assigned to either the study group or the control group. The study group (n = 68 boys and n = 41 girls) consisted of children diagnosed with at least one of the following allergic diseases: AD, FA, or anaphylaxis. Inclusion criteria for this group required a positive sIgE result for at least one tested allergen (sIgE ≥ 0.3 kUA/L). The subgroup classifications were as follows: (1) children with the diagnosis of AD established by a physician and based on the Hanifin–Rajka criteria; (2) children with a definite diagnosis of IgE-mediated FA, understood as an adverse health reaction that consistently occurs upon exposure to a particular food; and (3) children diagnosed with IgE-mediated anaphylaxis after exposure to certain allergen based on suggestive clinical history supported by elevated IgE antibody concentration [18,19] (Figure 1). Participants who did not meet the criteria for the study group but satisfied the control group criteria were assigned accordingly. The control group included children (n = 26 boys and n = 31 girls) with negative sIgE results for all tested allergens, no history of allergic diseases, and no family history of such conditions. All the children enrolled in the study were examined in order to verify the exclusion criteria. Key exclusion criteria included refusal to participate in the research, intellectual disability, allergen immunotherapy within the past 3 months, and biological treatment within the past 3 months.

### 2.3. Blood Sample Collection, Storage, and Preparation

Serum and whole blood samples for genetic analysis were collected during recruitment and prepared to store until the day of determination at −20 °C. The concentration of TSLP and IL-1β in serum were measured using a human TSLP/thymic stromal lymphopoietin ELISA KIT (Cat. No. E1320h, Wuhan EIAab Science, Wuhan, China) and an interleukin-1 beta ELISA KIT (Cat. No. E0563h, Wuhan EIAab Science) following the protocol provided by the manufacturer. The intensity of staining was quantified with a Biotek ELX800 plate reader (Cat. No. 6880G01EA Biotek, Vinooski, VT, USA) and reported in pg/mL.

DNA was extracted from EDTA whole blood samples utilizing a QIAamp DNA Blood Mini Kit (Cat. No. 51104 Qiagen GmbH, Hilden, Germany). The specimens from all participants were analyzed for specific SNPs, namely TSLP rs2289277 and IL-1β rs16944 (C-511T). All SNPs were identified using real-time polymerase chain reaction (PCR) assays followed by melting curve analysis with SimpleProbe^®^ probes (TibMolbiol, Berlin, Germany; Reference (NCBI 08/2020): https://www.ncbi.nlm.nih.gov/snp/rs16944; accessed on 5 January 2023) Reference (NCBI 12/2022): https://www.ncbi.nlm.nih.gov/snp/rs2289277; accessed on 5 January 2023), designed to complement the wild-type sequences. PCR was conducted at a final volume of 20 µL containing 1 µL of DNA at a concentration of 10–50 ng/µL, 1 µL of reagent mix containing specific primers and SimpleProbes^®^ probes at optimized concentration, 1.6 µL of MgCl_2_, and 2 µL of LightCycler^®^ FastStart DNA MasterHybProbe (Roche Applied Science, Mannheim, Germany). The experiments were conducted on a LightCycler 1.5 platform (Roche Applied Science, Mannheim, Germany). Negative controls were incorporated into each reaction to ensure the quality of every step in the genotyping process.

### 2.4. Statistical Analysis

A database was established with data obtained from questionnaires and laboratory analysis results and subjected to statistical analysis. The calculations were carried out using analytics software STATISTICA v.13.3 (TIBCO Software Inc., Woy Woy, NSW, Australia) and Excel (Microsoft^®^ Excel^®^, Microsoft 365 MSO version number 2403, 16.0.17425.20176). For all quantitative traits, descriptive statistics were assessed. To verify the compatibility of empirical distributions with the theoretical normal distribution, the Shapiro–Wilk test was applied. Qualitative features are presented in the tables as frequencies (n) and percentages (%). For all tests, *p*-values below 0.05 were regarded as statistically significant. The Kruskal–Wallis test for one-way analysis of variance by ranks was conducted to compare the results between the different subgroups. The Hardy–Weinberg equilibrium was evaluated by performing the χ^2^ goodness-of-fit test, which compared frequencies of genotypes expected and observed in the control group. The χ^2^ test was used to evaluate differences in genotype distribution and demographic features between the case and control groups. Logistic regression analysis was used to determine the relationship between genotypes or alleles and patient groups compared to control subjects, calculating the odds ratio (OR), 95% confidence interval (95% CI), and *p*-values for crude ORs. Three distinct genetic models were individually tested when comparing genotypes and subgroups with single and multiple atopic phenotypes. The three genotype groups were examined separately, with wild-type homozygotes serving as the reference group to assess a co-dominant model. To evaluate a dominant model, wild-type homozygotes were contrasted with both heterozygotes and homozygotes for minor alleles. In the multiplicative model, genotypes were categorized into a three-level variable for the number of minor alleles. The non-parametric data were analyzed using the Mann–Whitney U test. Additionally, we used the Benjamini-Hochberg method with a cut-off point of 5% across all performed analyses to adjust the *p*-values for multiple comparisons to maintain the reliability and accuracy of the statistical results and thus improve the validity of conclusions.

## 3. Results

### 3.1. Characteristics of the Study Population

The clinical characteristics of the population analyzed in this study, along with the distribution of the TSLP rs2289277 and IL-1β C-511T (rs16944) genotypes, are summarized in Table 1. The patients and control group did not differ significantly in age, but atopic patients were more frequently boys.

The study population was comprehensively characterized with regard to atopic status, and the allergy profiles of the patients were thoroughly assessed. These data are presented in Table 2. The highest percentages of allergies in the study group were grass pollen, birch pollen, tree nuts, and peanuts. Most patients in the study group exhibited sensitivity to more than one but fewer than five tested allergens.

### 3.2. The Correlation of Serum Level of TSLP and IL-1β with Single and Multiple Atopic Diseases

**Figure 2:**
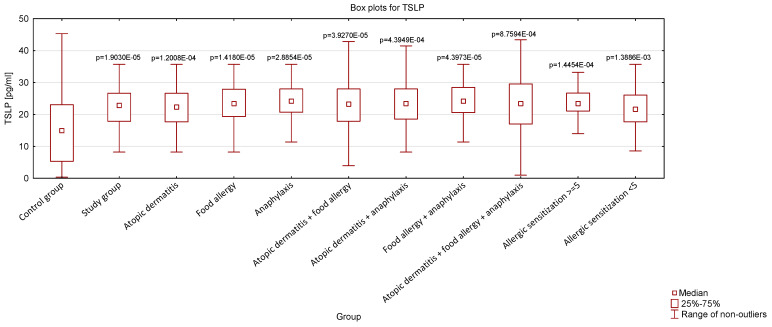
Serum concentrations of TSLP in the studied population. Statistical significance is indicated, with *p*-values displayed in exponential notation. The Kruskal–Wallis test was used for analysis, with a significance level set at *p* < 0.05. All results were compared to the control group.

**Figure 3:**
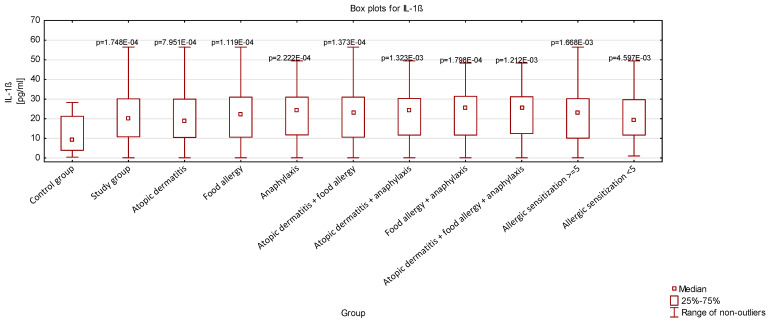
Serum concentrations of IL-1β in the studied population. Statistical significance is indicated, with *p*-values displayed in exponential notation. The Kruskal–Wallis test was used for analysis, with a significance level set at *p* < 0.05. All results were compared to the control group.

Comparing the concentrations of serum levels of TSLP and IL-1β, we demonstrated that the study group was characterized by higher levels of both cytokines compared to the controls. To further analyze the data, we divided the study group into subgroups based on the conditions they presented. We demonstrated that patients with AD, FA, and anaphylaxis exhibited significantly higher concentrations of the studied cytokines compared to the control group. Statistically, highly significant increases in serum TSLP and IL-1β levels were also observed in individuals with multiple allergic conditions (AD + FA, AD + anaphylaxis, FA + anaphylaxis, and AD + FA + anaphylaxis). Importantly, the results remained statistically significant after correction for multiple tests. The analysis of cytokine concentrations across these subgroups is shown in Figure 2 and Figure 3 (three extreme outlier values were excluded from the analysis to improve the clarity and readability of the graph). The study group was further divided into two subgroups: children sensitized to 1–5 allergens, and children sensitized to 5 or more allergens. Cytokine levels were analyzed across these subgroups and were found to be elevated compared to the control group. However, no significant differences were observed between these two subgroups.

### 3.3. The Association of TSLP SNP rs2289277 and IL-1β C-511T (rs16944) with Single and Multiple Atopic Diseases

The frequency of individual genotypes differed significantly across nearly all subgroups with defined diseases that we analyzed for the TSLP polymorphism compared to the control group. This trend was also observed in the overall study group, defined by a positive sIgE result for at least one tested allergen. Particularly, when we specifically analyzed the group with polysensitization, the frequency differences were found to be highly statistically significant (χ^2^ = 7.902; *p* = 0.019). In contrast, for the IL-1β C-511T (rs16944) polymorphism, no significant associations were observed regarding the frequencies of the genotypes. However, when analyzing the group of patients with all three conditions—AD, FA, and anaphylaxis, simultaneously—the differences in the distribution of genotypes were seen, although the statistical significance of this association was borderline (χ^2^ = 5.751; *p* = 0.056).

Analysis in the subgroup of patients with two comorbid allergic diseases revealed that the TSLP risk allele [C] was significantly associated with a more than twofold increase in susceptibility to the development of combined phenotypes, increasing the risk by up to 4–5-fold in patients carrying two alleles [C]. Using the codominant model, the OR associated with TSLP SNP was estimated at 3.94 (95%CI 1.34 ÷ 11.6) for AD combined with FA, at 4.89 (95%CI 1.48 ÷ 16.1) for AD combined with anaphylaxis, and at 4.85 (95%CI 1.55 ÷ 5.18) for patients with FA and anaphylaxis. Interestingly, the highest risk associated with TSLP rs2289277 was observed in patients with all three conditions, where the model showed a threefold increased risk for the allele-based analysis and a sevenfold increased risk in the codominant model—(OR = 7.34; 95%CI 1.96 ÷ 27.4; *p* = 0.004). These associations remained significant in both tested models even after accounting for multiple comparisons using the Benjamini-Hochberg, with a significance threshold of adj. *p* < 0.05. The statistical analysis outcomes are summarized in Table 3.

The analysis of the rs16944 SNP in the IL-1β gene revealed no significant associations with single or two coexisting atopic diseases examined in this study across any of the three genetic models analyzed independently. However, in patients with polysensitization and those diagnosed with all three studied allergic diseases, a trend towards a nearly significant increase in risk was observed in the allelic model. Notably, in the dominant model, we observed a statistically significant association between IL-1β SNP and the presence of all three studied conditions; a patient carrying at least one IL-1β rs16944 allele [T] was approximately three times more likely to have AD, AF, and anaphylaxis simultaneously (OR = 2.89; 95% CI 1.18 ÷ 7.08; *p* = 0.031) compared with the wild-type homozygote. However, the results did not reach statistical significance after correction for multiple tests.

### 3.4. The Association Between TSLP SNP rs2289277 and IL-1β C-511T (rs16944) and Serum Level of TSLP and IL-1β

To evaluate the influence of the investigated genetic variations on systemic expression levels of TSLP and IL-1β, we compared interleukin concentrations between individuals carrying the risk variants and those with the wild-type genotype (non-carriers) in the overall population; the analysis also took into account patients from specific subgroups. However, our analysis did not demonstrate any significant association between the presence of these polymorphisms and the circulating levels of TSLP and IL-1β, suggesting that these genetic variations may not directly influence the systemic expression of these cytokines in our study population (Table 4).

## 4. Discussion

The study findings indicate that the serum levels of TSLP and IL-1β were significantly elevated in patients with AD, FA, and anaphylaxis, as well as in individuals with multiple allergic conditions. Elevated cytokine levels were also observed in children sensitized to multiple allergens, supporting the role of these cytokines in promoting and sustaining allergic inflammation. Analysis of the TSLP SNP rs2289277 revealed that individuals carrying the risk allele [C] had nearly double the risk of developing allergic diseases, with homozygotes for the [C] allele showing a nearly threefold increased risk for AD, FA, and anaphylaxis. The presence of TSLP rs2289277 further increased the risk of having two concurrent allergic diseases, with the highest risk observed in patients with all three conditions, where the risk was sevenfold higher in the codominant model. For the IL-1β SNP (rs16944), no significant associations with allergic diseases were found, although a trend towards increased risk was observed in patients with polysensitization or all three allergic conditions. Our analysis did not show any significant impact of these genetic variations on the systemic expression levels of TSLP and IL-1β, suggesting that the polymorphisms may not directly influence cytokine concentrations in the study population. The study findings indicate that children with allergic diseases exhibit higher concentrations of both TSLP and IL-1β compared to healthy controls, supporting the role of the cytokines in promoting and sustaining allergic inflammation.

Elevated TSLP levels, consistent with prior research, highlight its function in allergic diseases. Similarly, IL-1β’s role as a potent pro-inflammatory mediator reinforces its significance in amplifying inflammatory pathways, contributing to disease chronicity and exacerbations.

A meta-analysis of studies evaluating TSLP concentrations in the blood of patients with atopic dermatitis revealed that serum TSLP levels are significantly elevated in individuals with AD compared to controls. Moreover, the analysis demonstrated that these levels are markedly higher in adults than in children [20]. However, certain studies have found that serum TSLP levels in AD patients are comparable to those in healthy controls [21]. In a study by Tsoi et al., elevated levels of TSLP were observed in the skin of patients with AD, with levels being significantly higher in chronic lesions compared to acute ones, emphasizing its role in enhancing the inflammatory response in AD [22].

We examined whether the coexistence of allergic diseases was associated with variations in cytokine concentrations; however, no such differences were identified within our study cohort. Nonetheless, Rothenberg-Lausell et al. revealed that in children with atopic AD and coexisting food allergies (AD FA+), nonlesional skin showed heightened TH2 activity compared to those with AD alone (AD FA−). The AD FA+ group demonstrated greater upregulation of TH2-related receptors, including IL-4R, CCR8, and the TSLP receptor (CRLF2). Additionally, they exhibited more severe skin barrier disruption, with increased transepidermal water loss, reduced filaggrin expression, and lower levels of key lipids. These findings highlight the role of elevated TH2 signaling, driven by factors like TSLP and impaired barrier function in the pathophysiology of AD with food allergies [23].

Kordulewska et al. found that children with allergies had significantly elevated levels of several cytokines, particularly IL-1β. In the allergy group, IL-1β levels were 5.5 times higher than in the control group, with a mean concentration of 337 pg/mL compared to 62 pg/mL in controls. Authors suggest that IL-1β could be a valuable biomarker for improving the diagnosis of allergic conditions [24]. Similarly, elevated IL-1β levels have been observed in other studies conducted on children with atopic conditions. However, considering the presence of multimorbidity, different outcomes were reported. In an analysis by Packi et al., IL-1β levels were significantly higher in patients with AD and IgE-mediated FA compared to patients with AD without FA or those with AD and delayed-type FA. Authors suggest that IL-1β could serve as a potential biomarker for IgE-mediated FA in children with AD [25].

A comparable conclusion regarding IL-1β as a biomarker for atopic diseases was drawn in a study conducted on 3097 children, which demonstrated that IL-1β could serve as a marker for active allergic conditions, including allergic rhinitis, asthma, and atopy [11].

Interestingly, our subgroup analyses revealed no significant differences in cytokine concentrations between children sensitized to 1–5 allergens and those sensitized to 5 or more allergens. This finding suggests that the overall cytokine milieu may be indicative of allergic status but does not necessarily correlate with the extent of sensitization. This emphasizes the complex interplay between immune mediators and clinical phenotypes of allergic diseases, warranting further exploration.

The analysis of the TSLP SNP rs2289277 polymorphism revealed a significant association between the risk C allele and increased susceptibility to specific allergic diseases, such as AD, FA, and anaphylaxis, with a higher risk in homozygous CC compared to the allele model. 

Additionally, the C allele was strongly associated with complex phenotypes, such as AD + FA, AD + anaphylaxis, and FA + anaphylaxis, and the combined phenotype of AD + FA + anaphylaxis, where the risk was elevated both in the allele and homozygous CC models. The presence of the C allele could contribute to a genetic predisposition that affects multiple allergic and immune responses simultaneously. Complex phenotypes often involve interactions between different genetic factors, environmental exposures, and immune system dysregulation, and the C allele may act as a modifier gene that increases the susceptibility to these overlapping conditions. This suggests that the C allele may play a broader role in the development of multiple allergic diseases, especially when they co-occur, rather than influencing each disease in isolation.

Our analysis of the SNP in the IL-1β gene showed no significant correlations with the atopic diseases studied, regardless of the genetic models tested individually. Likewise, in an Iranian cohort study, no significant association was observed between IL-1β-511C/T allele or genotype frequencies and AD. The authors emphasize the complexity of AD pathogenesis and the need for further research, particularly on gene-environment and gene-gene interactions, to elucidate the potential roles of IL-1β polymorphisms across diverse populations [26].

The outcomes of our study revealed no evidence of a significant relationship between the examined polymorphisms and the circulating concentrations of TSLP and IL-1β. Each group was analyzed separately, and no differences in cytokine levels were found between carriers of the risk allele and non-carriers, nor in comparisons between the dominant and co-dominant models.

Murrison et al. investigated the relationship between TSLP genetic variations, mRNA expression, circulating TSLP levels, and AD and asthma outcomes. The analysis focused on carriers with at least one copy of the risk allele for TSLP rs2289277 or rs11466750 compared to noncarriers with no copies of the risk alleles for these SNPs. They also showed that total TSLP circulating in plasma did not significantly differ between carriers and noncarriers, suggesting that systemic TSLP concentrations may not directly reflect genetic predisposition. In this study, elevated TSLP mRNA expression in the skin was observed in patients with AD. It is possible that serum cytokine levels are not always the most reliable indicator for assessing cytokine concentration. While no association was observed between the polymorphism and cytokine levels in the blood in our study, it is possible that a relationship might emerge when examining cytokine expression in the skin, which could offer deeper insights into the pathogenesis of AD [14]. Polymorphisms might influence the expression of TSLP or IL-1β in tissue-specific contexts, such as skin or mucosal tissues, rather than in circulating blood. Additionally, these polymorphisms could interact with other genetic variants or environmental factors, contributing to the multifactorial nature of the investigated allergic conditions.

Our study has several strengths that contribute to its significance in understanding allergic diseases. One notable advantage is the comprehensive approach, which integrates the analysis of both cytokine levels and genetic factors. This dual focus provides valuable insights into the potential interplay between immune mediators and genetic predisposition in the pathogenesis of allergic conditions. There is a scarcity of studies analyzing the impact of these specific SNPs on allergic diseases, making our investigation particularly valuable in contributing to this underexplored area. The use of a standardized protocol across all participants ensured consistency and reliability of data collection, allowing for meaningful comparisons between atopic children and healthy controls. Additionally, by analyzing a wide atopic profile using molecular analyses, this study offers a more nuanced understanding of atopic multimorbidity, laying the groundwork for future investigations into cytokines as potential biomarkers and their role in disease progression. However, the study also has limitations that should be addressed in future research. The absence of severity scoring for AD using tools like the SCORing Atopic Dermatitis Index (SCORAD) limits the ability to correlate cytokine levels with disease severity. Furthermore, insufficient data on recent allergen exposures and the intensity of these exposures may have impacted the interpretation of cytokine levels. Details about the timing and nature of the most recent anaphylaxis episodes were also limited, with only a minimum interval of eight weeks known prior to recruitment. The study relied on single-point cytokine measurements, which do not account for intrasubject variability or the potential influence of acute exposures, and it did not include local cytokine measurements in the skin, which might better reflect localized immune responses. In our study, we focused on the pediatric population; however, it would be valuable to extend future research to include adult cohorts to determine whether similar findings are observed across different age groups. Lastly, allergic respiratory conditions such as asthma and allergic rhinitis, important components of the atopic spectrum, were not analyzed, leaving a gap in the broader understanding of allergic diseases. Despite these limitations, the findings provide a solid foundation for future studies aiming to explore the complex interactions between genetic predisposition, cytokine regulation, and the clinical manifestations of allergic diseases. Considering the intricate associations between childhood allergic diseases and the atopic march, it is imperative to undertake studies that comprehensively integrate clinical manifestations, a rigorously defined atopic profile, the involvement of specific cytokines, and the underlying genetic predisposition.

## 5. Conclusions

The genetic analysis conducted in this study enhances the understanding of allergic multimorbidity by examining polymorphic variations in TSLP and IL-1β genes, particularly TSLP rs2289277 and IL-1β rs16944. While no significant differences in genotype frequencies were found for IL-1β rs16944, notable associations were observed for TSLP rs2289277. Carrying a higher number of the risk allele (C) in the TSLP SNP rs2289277 is associated with the greatest likelihood of having multiple concurrent allergic diseases, with the highest risk observed in individuals with all three conditions—AD, AF, and anaphylaxis—simultaneously. Moreover, individuals carrying the risk allele were more likely to be sensitized to multiple allergens, with this likelihood further increased in those with two copies of the risk allele. Additionally, serum levels of TSLP and IL-1β were higher in the study groups compared to the control group, underscoring their relevance to disease pathogenesis. Although SNPs can influence gene expression, this does not always correlate with altered cytokine levels in the bloodstream. Complex mechanisms, possibly involving multiple genetic variants and environmental factors, likely drive elevated cytokine concentrations. Further research is needed to clarify these pathways and their contribution to allergic disease development, with longitudinal studies offering insights into disease progression and potential for personalized therapies.

## Figures and Tables

**Table 1:** Characteristics of the study population.

Characteristic	Study Group n = 109	Control Group n = 57
Gender, n (%):		
Girls	41 (37.61%)	31 (54.39%)
Boys	68 (62.39%)	26 (45.61%)
Age at the time of recruitment (years):		
M ± SD	6.59 ± 4.74	6.59 ± 5.78
Me [Q1; Q3]	5 [3.00;10.00]	4 [1.67;12.00]
Min–Max	0.7–17	0.33–17
Positive family history of allergy:		
Yes	77 (70.64%)	5 (8.77%)
No	32 (29.36%)	52 (91.23%)
Number of siblings:		
M ± SD	0.84 ± 0.71	0.88 ± 0.96
Me [Q1; Q3]	1 [0;1]	1 [0;1]
Min–Max	0–3	0–9
Specific SNP—TSLP rs2289277		
CC	37 (33.94%)	11 (19.30%)
CG	52 (47.71%)	29 (50.88%)
GG	20 (18.35%)	17 (29.82%)
Specific SNP—IL-1β C-511T rs16944		
TC	52 (47.71%)	24 (42.11%)
CC	50 (45.87%)	30 (52.63%)
TT	7 (6.42%)	3 (5.26%)

**Table 2:** Atopic status of the study group. The study group included atopic patients, defined as those diagnosed with AD, FA, or anaphylaxis and having a positive sIgE result for at least one tested allergen (sIgE ≥ 0.3 kUA/L).

Outcomes	(Number, %):
Atopic dermatitis	85 (77.98%)
Food allergy	78 (71.56%)
History of anaphylaxis	57 (52.59%)
Atopic dermatitis and food allergy	54 (49.54%)
Atopic dermatitis and anaphylaxis	42 (38.53%)
Food allergy and history of anaphylaxis	51 (46.79%)
Atopic dermatitis, food allergy, and anaphylaxis	36 (33.02%)
Sensitization	(number, %)
Peanuts	45 (41.28%)
Tree nuts	53 (48.62%)
Soy	31 (28.44%)
Sesame	28 (25.93%)
Parvalbumins	13 (11.93%)
Cow milk	22 (20.18%)
Egg	22 (20.18%)
Wheat	13 (11.93%)
Shrimp	7 (6.42%)
Grass pollen	57 (52.29%)
Birch pollen	57 (52.29%)
Dust mites	29 (26.61%)
Alternaria spp.	22 (20.18%)
Lipocalins (dog or cat)	40 (36.70%)
Polysensitization	(number, %)
≥1 allergen and <5	65 (59.63%)
≥5 allergens	44 (40.37%)

**Table 3:** Risk assessment for allergic diseases with respect to the analyzed SNPs. A *p*-value of <0.05 was considered statistically significant. * Adjusted *p*-value refers to the *p*-value after the Benjamini–Hochberg correction, FDR < 0.05.

Phenotype	Total *n* (%)	TSLP rs2289277				IL-1β rs16944			
		Allele C Counts	Risk Allele C Frequency (%)	G vs. C *p*-Value OR (95% CI)	G vs. C Adjusted *p*-Value *	Allele T Counts	Risk Allele T Frequency (%)	C vs. T *p*-Value/ OR (95% CI)	C vs. T Adjusted *p*-Value *
Control group	57/166 (34.3%)	51/114	44.74%	Ref.	Ref.	30/114	26.32%	Ref.	Ref.
Atopy/allergic sensitization	109/166 (65.6%)	126/218	57.80%	*p* = 0.028 1.69 (1.07 ÷ 2.67)	*p* * = 0.032	66/218	30.28%	*p* = 0.524 1.21 (0.73 ÷ 2.02)	*p* * = 0.693
Atopy/allergic sensitization ≥ 5	44/109 (40.4%)	56/88	63.64%	*p* = 0.010 2.16 (1.22 ÷ 3.82)	*p* * = 0.022	33/88	37.5%	*p* = 0.095 1.69 (0.92 ÷ 3.06)	*p* * = 0.427
Atopic dermatitis	85/166 (51.2%)	102/170	60.00%	*p* = 0.015 1.85 (1.15 ÷ 2.99)	*p* * = 0.027	52/170	30.59%	*p* = 0.505 1.23 (0.73 ÷ 2.09)	*p* * = 0.318
Food allergy	78/166 (46.9%)	92/156	58.97%	*p* = 0.026 1.78 (1.10 ÷ 2.89)	*p* * = 0.033	53/156	33.97%	*p* = 0.185 1.44 (0.84 ÷ 2.45)	*p* * = 0.331
Anaphylaxis	57/166 (34.3%)	70/114	61.4%	*p* = 0.017 1.69 (1.16 ÷ 3.33)	*p* * = 0.025	39/114	34.21%	*p* = 0.249 1.46 (0.82 ÷ 2.57)	*p* * = 0.333
Atopic dermatitis + food allergy	54/166 (32.5%)	68/108	62.96%	*p* = 0.007 2.10 (1.23 ÷ 3.59)	*p* * = 0.021	39/108	36.11%	*p* = 0.147 1.58 (0.89 ÷ 2.81)	*p* * = 0.373
Atopic dermatitis + anaphylaxis	42/166 (25.3%)	55/84	65.48%	*p* = 0.004 2.34 (1.31 ÷ 4.20)	*p* * = 0.018	29/84	34.52%	*p* = 0.271 1.48 (0.80 ÷ 2.73)	*p* * = 0.348
Food allergy + anaphylaxis	51/166 (30.7%)	66/102	64.71%	*p* = 0.004 2.26 (1.31 ÷ 3.92)	*p* * = 0.018	38/166	37.25%	*p* = 0.106 1.66 (0.93 ÷ 2.96)	*p* * = 0.568
Atopic dermatitis + food allergy + anaphylaxis	36/166 (21.7%)	51/72	70.83%	*p* = 0.001 3.00 (1.60 ÷ 5.62)	*p* * = 0.009	28/72	38.89%	*p* = 0.071 1.782 (0.95 ÷ 3.35)	*p* * = 0.524

**Table 4:** Association between the TSLP SNP rs2289277 and IL-1β C-511T (rs16944) polymorphisms and serum concentrations [pg/mL] of TSLP and IL-1β is presented for each group. Statistical significance was determined using the Mann–Whitney U test with continuity correction. A *p*-value of <0.05 was considered statistically significant.

Phenotype	TSLP rs2289277			IL-1β rs16944		
	CG + CC	GG	*p*-Value	TC + TT	CC	*p*-Value
Control group	15.71	15.73	0.72	20.55	16.96	0.74
Study group	22.43	26.90	0.85	24.35	29.12	0.33
Allergic sensitization ≥ 5 allergens/study group	21.90	25.07	0.62	22.87	22.57	0.86
Atopic dermatitis	22.43	27.42	0.80	24.63	29.87	0.17
Food allergy	22.54	25.50	0.55	23.66	22.86	0.60
Anaphylaxis	22.77	26.17	0.54	24.06	24.34	0.82
Atopic dermatitis + food allergy	22.56	24.94	0.76	23.94	21.85	0.51
Atopic dermatitis + anaphylaxis	22.10	26.12	0.44	23.60	21.69	0.74
Food allergy + anaphylaxis	22.77	26.88	0.43	23.89	25.66	0.90
Atopic dermatitis + food allergy + anaphylaxis	22.11	27.32	0.35	23.37	22.61	0.93

## Data Availability

The original contributions presented in this study are included in the article. Further inquiries can be directed to the corresponding author.
